# Towards reliable healthcare Imaging: conditional contrastive generative adversarial network for handling class imbalancing in MR Images

**DOI:** 10.7717/peerj-cs.2064

**Published:** 2024-07-10

**Authors:** Lijuan Cui, Dengao Li, Xiaofeng Yang, Chao Liu

**Affiliations:** 1 College of Computer Science and Technology (College of Data Science), Taiyuan University of Technology, Taiyuan, Shanxi, China; 2 Department of Urology, First Hospital of Shanxi Medical University, Taiyuan, Shanxi, China; 3 School of First Hospital of Shanxi Medical University, Taiyuan, Shanxi, China

**Keywords:** Segmentation, MRI images, Classification, Generative adversarial network, Machine learning, Medical imaging

## Abstract

**Background:**

Medical imaging datasets frequently encounter a data imbalance issue, where the majority of pixels correspond to healthy regions, and the minority belong to affected regions. This uneven distribution of pixels exacerbates the challenges associated with computer-aided diagnosis. The networks trained with imbalanced data tends to exhibit bias toward majority classes, often demonstrate high precision but low sensitivity.

**Method:**

We have designed a new network based on adversarial learning namely conditional contrastive generative adversarial network (CCGAN) to tackle the problem of class imbalancing in a highly imbalancing MRI dataset. The proposed model has three new components: (1) class-specific attention, (2) region rebalancing module (RRM) and supervised contrastive-based learning network (SCoLN). The class-specific attention focuses on more discriminative areas of the input representation, capturing more relevant features. The RRM promotes a more balanced distribution of features across various regions of the input representation, ensuring a more equitable segmentation process. The generator of the CCGAN learns pixel-level segmentation by receiving feedback from the SCoLN based on the true negative and true positive maps. This process ensures that final semantic segmentation not only addresses imbalanced data issues but also enhances classification accuracy.

**Results:**

The proposed model has shown state-of-art-performance on five highly imbalance medical image segmentation datasets. Therefore, the suggested model holds significant potential for application in medical diagnosis, in cases characterized by highly imbalanced data distributions. The CCGAN achieved the highest scores in terms of dice similarity coefficient (DSC) on various datasets: 0.965 ± 0.012 for BUS2017, 0.896 ± 0.091 for DDTI, 0.786 ± 0.046 for LiTS MICCAI 2017, 0.712 ± 1.5 for the ATLAS dataset, and 0.877 ± 1.2 for the BRATS 2015 dataset. DeepLab-V3 follows closely, securing the second-best position with DSC scores of 0.948 ± 0.010 for BUS2017, 0.895 ± 0.014 for DDTI, 0.763 ± 0.044 for LiTS MICCAI 2017, 0.696 ± 1.1 for the ATLAS dataset, and 0.846 ± 1.4 for the BRATS 2015 dataset.

## Introduction

Medical image segmentation plays a crucial role in clinical applications, allowing the precise description of anatomical structures and pathological regions, aiding diagnosis, treatment planning, and disease monitoring. Accurate segmentation of medical images has foremost importance, as it directly impacts the quality of medical decision-making and care. The persistent challenge in this field involves dealing with imbalanced data, where the majority of pixels correspond to healthy regions, and the minority represent non-healthy areas. This class imbalance creates a significant hurdle to achieve reliable and unbiased segmentation performance because models tend to favour the more prevalent class, compromising sensitivity and clinical utility (Singh Rawat & Kumar Mishra, 2022). Imbalanced data distributions are common in medical imaging due to the inherent scarcity of certain pathological conditions or anatomical structures of interest. Conventional machine learning and deep learning networks trained on such datasets often struggle to generalize well, resulting in poor performance in the minority classes. Inaccurate segmentation can have severe consequences, including incorrect disease diagnoses, inaccurate treatment plans, or interventions, which can impact patient outcomes (Litjens et al., 2017). Recently, researcher pay more attention on addressing class imbalancing challenge during the disease classification, tumour localization, and segmentation. Researchers have introduced two main approaches to tackle this issue: (1) data-level strategies and (2) algorithm-level strategies. In the data-level strategies (Fotouhi, Asadi & Kattan, 2019), the primary objective is to rectify class imbalances by adjusting the distribution of the dataset. This data adjustment can be achieved by various methods, such as the Synthetic Minority Over-sampling Technique (SMOTE) for augmenting the positive class (Douzas & Bacao, 2018; Mariani et al., 2018) or reducing the negative class through under-sampling (Jang et al., 2014a). However, one drawback of data adjustment is that it may sometime lead to the removal of crucial samples or the addition of redundant samples to the training set. Furthermore, alternative approaches involve iterative sampling (Rodriguez et al., 2012) and gradually refining mini batches for training deep neural networks (Dong, Gong & Zhu, 2019). The algorithm-level strategies (Fernández et al., 2018) aim to address this issue of class imbalance by adjusting the learning weights of the algorithm, which helps to reduce the bias toward the majority class. Examples of these modifications include the use of loss functions like accuracy loss (Sudre et al., 2017), Dice coefficient loss (Isensee et al., 2018b; Isensee et al., 2018a), and asymmetric similarity loss (Salehi, Erdogmus & Gholipour, 2017), which adjust the learning process by assigning different costs to misclassifying instances of the minority and majority classes. It is important to note that these loss functions may address specific aspects of application quality. For instance, additional measures like mean surface distance or Hausdorff surface distance might be necessary for segmentation tasks. Another set of approaches focus on achieving balance through ensemble learning, where similar or dissimilar classifiers are combined to enhance their generalization capabilities. In Sun et al. (2007a), the authors have studied the impact of combining redundant ensembles regarding bias and variance. Through ensemble modelling, the predicted results in the minority class tend to improve due to reduced variance. In this work, we aim to address the adverse effects of class imbalance by integrating generative adversarial networks (GAN) with contrastive learning techniques. The proposed research is based on a conditional generative network for medical image segmentation, signifying its effectiveness in the liver lesion, breast lesion, thyroid, and brain tumour segmentation tasks (Rezaei et al., 2019).

Recent studies (Yeung et al., 2021; Sharma, Kiros & Salakhutdinov, 2015; Ding et al., 2020; Li et al., 2022; Oktay et al., 2018) integrated attention mechanisms and dedicated loss functions (Taghanaki et al., 2019; Cui et al., 2019; Quan, Nguyen-Duc & Jeong, 2018; Dou et al., 2018) to address class imbalance. However, existing loss functions often rely on class frequency for determining class importance, limiting their effectiveness. Likewise, attention-based approaches focus on channel-wise or spatial information, overlooking the relationship between channels and classes. Class-wise attention handles this by extracting distinctive features for each class. Several studies have explored the pixel rebalance methods (Pan et al., 2023; Hong et al., 2021) to tackle class imbalance. However, this approach suffered from training inconsistencies and decrease in segmentation performance. Pixel rebalance relies on class frequency as a guiding principle. This is problematic because pixels within an image are not independent and identically distributed like the samples in a dataset. Pixels often exhibit spatial correlations, making class frequency in the pixel domain an unreliable indicator for rebalancing. To address this limitation, we have adopted a region rebalance approach. This method rebalances pixels by grouping them into regions, leading to a more robust solution for handling class imbalance. In medical imaging accurate segmentation is very crucial for diagnosis and treatment planning. CGANs are selected in medical imaging due to their ability to incorporate additional information into the generative process. This additional conditioning helps the generative process, capturing subtle and contextual patterns within images and makes the model more adaptive and tailored to the specific requirements of the segmentation task. The proposed conditional contrastive GAN(CCGAN) addresses limitations introduced due to class imbalance by introducing a class-specific attention mechanism for better ROI identification, a region rebalancing mechanism for robustness across lesion sizes, and supervised contrastive based learning network (SCoLN) to mitigate misclassification costs.

The contribution of the designed network is explained in the following:

1. We have developed a novel class-specific attention mechanism, which excels at identifying regions of interest (ROI) based on class-distinguishing features.
2. We have integrated a region rebalancing mechanism to improve the robustness of the proposed model to different regions and sizes of lesions.
3. Our proposed network utilizes supervised contrastive learning to tackle the challenges posed by imbalanced data and to mitigate the high cost of misclassification, especially crucial in medical applications.

The rest of the article is organized as follows: “Literature Review” is a review the literature, “Methodology” presents proposed methodology, “Dataset” provides results analysis and comparison, and “Result Analysis and Discussion” the “Conclusion” discusses and concludes this work.

## Literature Review

Recently, several studies have focused on the class and data imbalance problem for segmentation and classification using various deep-learning models. This section summarizes the most relevant works in the field, highlighting their contributions, strengths, and limitations. A significant challenge in medical image analysis is handling imbalanced data, a problem that is particularly acute in the field of medical imaging. For instance, in tasks like thyroid tumour segmentation, the size of the normal thyroid region typically exceeds that of the abnormal region. Training segmentation networks with such imbalanced data can lead to instability and bias towards the class with the larger region. Three primary strategies are employed to address class imbalance: over-sampling the positive class (Douzas & Bação, 2018), under-sampling the negative class (Jang et al., 2014b), and using SMOTE (Chawla et al., 2002), which creates synthetic data along the line segments connecting minority class samples. While these methods are straightforward to implement, they may lead to the removal of crucial data or the addition of redundant data in the training set. Synthetic resampling techniques, particularly those based on SMOTE (Chawla et al., 2002), are often favored due to their simplicity and classifier-independent nature as preprocessing steps. Methods based on SMOTE create samples through random interpolation among the k-nearest neighbors in the minority class. Although these techniques have demonstrated enhanced performance in shallow models, they do not effectively improve calibration concerning underrepresented classes. In deep learning, it’s often impractical to load and preprocess the entire dataset at once. As resampling is conducted within each mini-batch, as suggested by Huang et al. (2016a). However, calculating k-nearest neighbors for synthetic oversampling in each mini-batch can significantly slow down training and may be unfeasible for large-scale problems. Furthermore, the synthetic distribution created by SMOTE has been observed to compress the minority class distribution, as noted by Elreedy & Atiya (2019). This compression limits the possible reduction in predictive bias, a problem that becomes more pronounced in mini-batches where only a portion of minority samples may be presented. Additionally, SMOTE can adversely affect the class distribution, especially in scenarios involving disjoint data distributions, noise, and outliers, as identified by Bellinger et al. (2020). Recent strategies developed to address class imbalance in deep learning have emphasized creating extra synthetic samples using GANs and VAEs (Variational Autoencoders) to equalize the training set (Mullick, Datta & Das, 2019; Dai et al., 2019). These techniques are primarily used in image classification and require the training of additional models and/or a substantial increase in parameters. Such training processes are resource-intensive and also prone to issues with imbalanced classes and scarce training examples.

 Kamnitsas et al. (2017) proposed a patch-based strategy to mitigate the class imbalance issue. In this approach, training patches are extracted with a 50 percent chance of being centered either on lesioned or healthy voxels. Havaei et al. (2017) suggested a two-stage training method. Initially, it creates a patch dataset ensuring equal probability for all labels by considering the variety in all classes. Subsequently, it retrains solely the output layer to accurately adjust the output probabilities. This approach effectively addresses the class imbalance issue. Cì et al. (2020) employed a lesion-centered approach, where all training patches are derived from areas centered around a lesion voxel. To prevent location bias, where a lesion voxel is consistently anticipated at the patch center, they introduced a random offset to the sampling point. This technique also contributes to data augmentation.

Positional contrastive learning (PCL) framework was proposed by Zeng et al. (2021), innovatively addresses the challenge of generating effective contrastive pairs in medical image segmentation. They incorporate positional information, which is a basic step for adapting contrastive learning in medical image processing. The proposed PCL method effectively work on the CT and MRI datasets, demonstrating its superior performance in both semi-supervised and transfer learning scenarios. Another approach to unsupervised domain adaptation (UDA) for medical image segmentation was developed by Liu et al. (2022) named as margin preserving self-paced contrastive learning (MPSCL) model. This method not only aligns category-aware distributions more effectively through a novel margin preserving contrastive loss but also utilizes progressively refined pseudo-labels to strengthen the supervision in contrastive learning. Federated Contrastive Learning (FCL) (Wu et al., 2022) framework proficiently addresses the challenge of limited data diversity in federated learning for medical image segmentation. They used the exchanging features strategy during the pre-training process to enhance the effectiveness of contrastive learning across different sites without compromising data privacy. Another work performed by Liu, Aviles-Rivero & Schonlieb (2023) innovatively combines image-level registration with feature-level contrastive learning within a convolutional neural network framework for medical image segmentation. By capturing image-to-image transformation mappings and enhancing discriminative capacity through contrastive learning. Cyclical contrastive random walks (CCRW) approach was used by Fischer et al. (2023) to the medical imaging domain for reducing the need for large annotated datasets in semantic segmentation tasks. By focusing on inherent anatomical similarities and employing a multi-level supervision strategy for both local and global anatomical structures, this technique effectively controls the limited annotations, showing a notable improvement in the Dice Similarity Coefficient across MRI datasets.

 Zhang et al. (2019) suggested a mask-oriented hierarchical learning approach for segmenting breast tumours through fully convolutional networks (FCN) from DCE-MR images. They designed a dual-stage FCN model for the segmentation of breast tumours in two phases. They introduced reinforcement sampling technique and dice-sensitivity loss to address the issue of class imbalance. Sensitivity introduces an extra bias towards the identification of tumour pixels, thereby addressing the imbalance issue by shifting emphasis towards the minority class. Bria, Karssemeijer & Tortorella (2014) developed a method for computer-aided detection that tackles the class imbalance issue for breast cancer segmentation using a series of boosting classifiers. They employed a boosting technique at each node to handle the issue of class imbalancing. Each node in this series is trained using a learning algorithm that focuses on ranking rather than classification error.

Tao et al. (2022) introduced LCA-Net for segmenting thyroid nodules from MRI images. This network combines local features extracted by a convolutional neural network (CNN) with global features learned through a transformer network, which includes a contextual attention module. To address class imbalance, they incorporated an optimized loss function.

Some studies involve adopting a multi-task segmentation approach (Chen et al., 2018; Shen et al., 2017; Wang et al., 2018), which simplifies the complex multi-class segmentation task by dividing it into several easier tasks. In this approach, each training task focuses on segmenting just one region, resulting in a more balanced label distribution compared to segmenting multiple classes simultaneously. Some methods (Sun et al., 2007b) tackle the class imbalance issue by utilizing ensemble learning, which involves merging identical or varied classifiers to enhance their overall ability to generalize. Contrary to simple models, applying cost adjustment or resampling during the training of complex models lead to overfitting the limited information available in the minority classes. Anand et al. (1993) investigated the effects of training deep neural networks with imbalanced classification data for two class problem and discovered that errors from the majority class predominantly influence the gradient-based weight updates during the training process. Adjusting costs or resampling instances in the mini-batch are common methods for addressing bias in the case of CNN (Huang et al., 2016b; Cui et al., 2019). Consequently, these approaches might only enhance predictive accuracy in cases with slight to moderate imbalance. Furthermore, these methods do not better the calibration as they produce samples within the manifold and use definite labels for training.

## Methodology

### Overview

**Figure 1:**
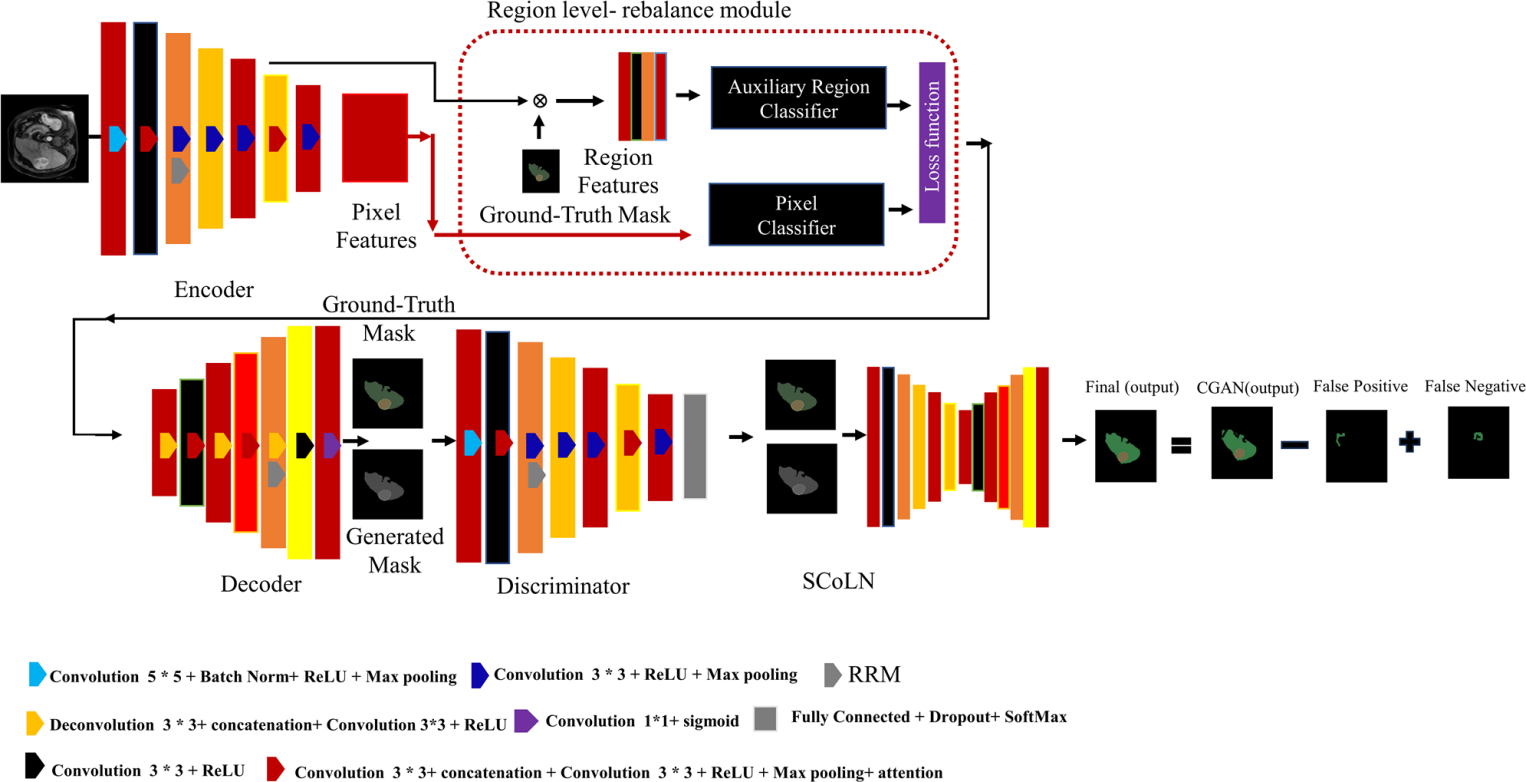
Typical conditional generative adversarial network. An overview of the CCGAN. The image and mask are provided as input, with the mask serving as a training reference to guide the generator in producing realistic outputs. The CCGAN comprises a generator, discriminator, and SCoLN. The generator produces segmented images, while the discriminator distinguishes between generated and real images. The SCoLN receives the output from the discriminator and generates the final output mask by incorporating false negatives and subtracting false positives.

In this section, we introduce the Conditional Contrastive Generative Adversarial Network (CCGAN) designed specifically for medical image segmentation. Our model addresses the challenges posed by class imbalance and pixel misclassification commonly encountered in medical imaging datasets. This research employs a conditional generative adversarial network (CGAN) augmented with contrastive learning as shown in Fig. 1. The proposed GAN based architecture highlights the innovative 2C loss concept (Chen et al., 2018) with the fusion of conditional approach with GAN. Similar to classic GAN training procedures, the CCGAN comprises three primary phases: (1) Generator training, (2) discriminator training and (3) SCoLN training. Generator and discriminator training focus on adversarial loss computation. A critical feature of this process is the calculation of 2C loss, which is done using ‘R’ real images during the discriminator’s training phase and ‘G’ generated images during the generator’s phase. To address the issue of misclassification costs, the discriminator’s output is passed to the supervised contrastive learning-based network (SCoLN), which is trained to thoroughly understand the erroneous predictions made by the CCGAN, including both false positives and false negatives. This approach allows the CCGAN to refine its performance by reducing the disparity in the embeddings of real images from the same class while increasing it for others. The addition of a class-specific attention module refines feature focus for improved accuracy, emphasizing specific channels essential for identification of distinct classes. Furthermore, the region-level rebalance module addresses class imbalance at a regional level, promoting a balanced segmentation process.

### Generative adversarial networks: background

In a generative adversarial network (GAN), the primary goal of the Generator G is to acquire the ability to map a random noise vector *n* to generate an output image O, which can be expressed as G: N → O. In parallel, the discriminator network, represented as D, calculates the probability of a given sample originating from the actual training data, *a*_*Real*_, rather than being generated by the Generator *a*_*Fake*_. The objective function of the typical GAN encapsulates the essence of a two-player minimax game, as demonstrated in Eq. (1).

(1) \begin{eqnarray*}{\text{Mini}}_{G}{\text{Max}}_{D}S \left( D,G \right) ={E}_{o}[\text{logD}(o)]+{E}_{a,n}[\log \nolimits (1-\mathrm{D}(\mathrm{G}(a,n)))]\end{eqnarray*}

where $S \left( D,G \right) $ represents the adversarial function between *D* and *G*, *Mini*_*G*_ minimization with respect to the generator (G) and *Max*_*G*_ the maximization with respect to the discriminator (D). *E*_*o*_[logD(*o*)] corresponds to the expectation over the real data (*o*) where the discriminator aims to maximize the probability of correctly classifying real data as real. The term *E*_*a*,*n*_[log(1 − D(G(*a*, *n*)))] corresponds to the expectation over the generated data G(*a*, *n*) where the discriminator aims to maximize the probability of correctly classifying generated data as fake.

The training scheme in classic CGAN for segmentation task operates similarly to a minimax game, as illustrated in Eq. (2).

(2) \begin{eqnarray*}{L}_{G}\leftarrow Min{i}_{G}Ma{x}_{D}S \left( D,G \right) ={E}_{a,{o}_{seg}}[\text{logD}(a,{o}_{seg})]+{E}_{a,n}[\log \nolimits (1-\mathrm{D}(a,G(a,n)))]\end{eqnarray*}

where [logD(*a*, *o*_*seg*_)] corresponds to the expectation over the real segmented data (*o*_*seg*_) and real input data (*a*) and *E*_*a*,*n*_[log(1 − D(*a*, *G*(*a*, *n*)))] corresponds to the expectation over the generated segmented data (G(a, n)) and real input data (*a*).

In this, the generator is responsible for producing segmented image, while the discriminator assesses both the ground truth data and the generator’s output to distinguish between real and synthetic segments.

### CCGAN network architecture

In the proposed CCGAN structure, we have employed a fully convolutional encoder–decoder network within the generator to generate labels for pixels. Similar to the UNet architecture (Liu et al., 2022), we have incorporated skip connections between each layer. The skip connections integrate all channels at each layer “l” with those at layer “t –l” where t represent total number of layers. In the encoder of the generator, we have employed a convolutional layer (5 × 5 kernel), batch normalization (BN) layer, Rectified linear unit (ReLU) as an activation function and down-sampling of 2 × 2 stride. The second block incorporates convolutional layers (3 × 3 filter), concatenation layer, BN and ReLU layers. The generator of the CCGAN also includes region rebalance module and class-specific attention module as depicted in Fig. 1. In the decoder part of the generator, we have implemented up-sampling through image resizing of factor 2 and a convolutional layer (3 × 3 kernel) with a stride of 1. In the proposed model’s, we concatenated the high-resolution features from multiple high-resolution images with up-sampled versions of global low-resolution features. This design choice enables the network to simultaneously learn both local and global feature. The discriminator is a fully convolutional network having the same structure as the encoder of the generator. The features extracted from the convolutional layers are subsequently passed to the SoftMax function to determine whether the segmented pixels corresponds to the true class.

#### Class-specific attention module

The suggested class-specific attention module is systematically utilized to enhance the multi-scale features of the fully convolutional network (FCN) across various levels, yielding refined attention maps. The choice of the level for attention allocation hinges on the high-level semantic content of the features. This approach has been implemented at the second and sixth levels of the proposed model. Following the application of attention layers, pooling and classification layers are employed to yield the final results.

**Figure 2:**
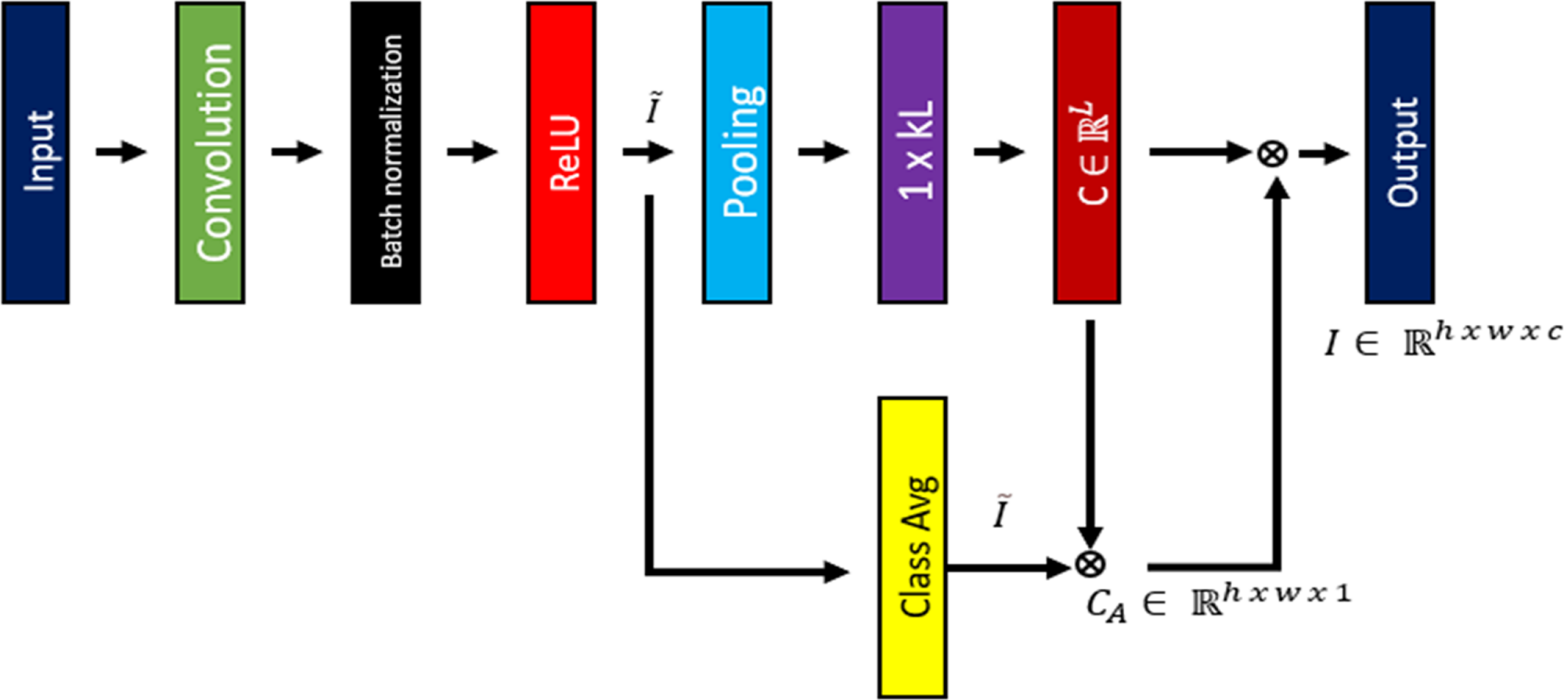
Synoptic overview of class attention mechanism.

To enhance the segmentation accuracy for specified classes, we have proposed a class-specific attention mechanism, as illustrated in Fig. 2. This approach is adept at identifying ROI using characteristics that differentiate between classes. The input representation from the encoder of the CCGAN is denoted as *I* ∈ ℝ^*h*×*w*×*c*^ = {*I*_1_, *I*_2_, …..*I*_*C*_ }, where h, w and c symbolizes height, width, and channel respectively. The input *I* goes through depth-wise separable convolution and subsequently batch normalization and rectified linear unit activation layers in order to get $\hat {I}$ ∈ ℝ^*h*×*w*×*c*^ = {$\widehat{{I}_{1}}$, $\widehat{{I}_{2}}$, …, $\widehat{{I}_{xL}}$ }. The whole process is mathematically formulized as shown in Eq. (3):

(3) \begin{eqnarray*}\hat {I}=ReLU(\alpha ({\partial }^{3\times 3}(I)))\end{eqnarray*}

where *α* indicates batch normalization and ∂ shows convolution operation. *x* denotes the number of channels necessary to ascertain feature maps capable of distinguishing among each class, while *L* signifies the total number of classes. A fundamental element of our proposed attention mechanism is the 3 × 3 depth-wise separable convolution operation, which enables our network to focus on specific channels crucial for identifying specified class.

Considering the input feature map $\hat {I}$, the score for each class is calculated by organizing the channels by class L, in accordance with the *x*, as in Eq. (4):

(4) \begin{eqnarray*}{C}_{i}= \frac{1}{x} \sum _{k=1}^{x}\rho \left( {\hat {I}}_{j,k} \right) ,j\in 1,2,L\end{eqnarray*}

where *C* = [*C*_1_, *C*_2_, *C*_3_, …, *C*_*L*_] represents class vector score, *ρ* denotes global maximum pooling. The class-specific semantic feature map, denoted as $\tilde {I}\in {\mathbb{R}}^{h\times w\times L}$ is derived by averaging the maps for each class as in Eq. (5):

(5) \begin{eqnarray*}\tilde {I}= \frac{1}{K} \sum _{j=1}^{x}{\hat {I}}_{j,k},j\in 1,2,\ldots ,L\end{eqnarray*}

The class-specific attention map, designated as *C*_*A*_, is derived as shown in Eq. (6).

(6) \begin{eqnarray*}{C}_{A}= \frac{1}{L} \sum _{i=1}^{L}C\tilde {I}\end{eqnarray*}

In the class-specific attention method, the regions that are particularly significant for ROI are emphasized. Subsequently, Eq. (7) employs this class-specific attention to the input feature map *I*

(7) \begin{eqnarray*}{I}_{{C}_{A}}=I\bigotimes {C}_{A}.\end{eqnarray*}

The output feature maps following class-specific attention, is represented as *I*_*C*_*A*__, where ⊗ denotes multiplication(element-wise).

#### Region-level rebalance module

In the context of MRI image segmentation, where the region of interest is typically small compared to the larger background, we have suggested a region-level rebalance (RR) training scheme that focuses on rebalancing at the region level rather than at the pixel level. This method addresses the challenge of class imbalance, particularly pertinent in scenarios with small target regions, by incorporating an auxiliary region classification module. This module promotes a more equitable distribution of features across different regions of input representation, ensuring a more balanced segmentation process. An advantage of this approach is that it does not incur extra computational costs during testing, as the region rebalancing module is excluded from the inference phase. The region rebalancing module consists of two main components: (i) an auxiliary region classification module, specifically designed to alleviate class imbalance problems in MRI segmentation where significant size disparities exist between regions of interest and the background. The auxiliary region classifier is comprised of input layers with dimensions 32 × 32 × C, where ’C’ denotes the number of channels. It incorporates convolutional layers with a 3 × 3 filter, ReLU activation, global average pooling, and a fully connected layer. And (ii) pixel classifier composed of convolution layers, batch normalization layers, activation and SoftMax layer. We have employed specific loss functions to facilitate this rebalancing process, thereby improving segmentation accuracy in MRI images with small areas of interest as depicted in Eqs. (8), (9) and (10).

(8) \begin{eqnarray*}& F \left( C \right) =\sum _{x\epsilon DS}1ifC\in {X}_{gt}otherwise0\end{eqnarray*}

(9) \begin{eqnarray*}& {L}_{reg}= \frac{1}{ \left\vert A \right\vert } \sum _{a\in A}-Log \left( \frac{F({a}^{y}){e}^{{A}_{y}}}{\sum _{k\in Y}F(k){e}^{{A}_{k}}} \right) \end{eqnarray*}

(10) \begin{eqnarray*}& {\text{Loss}}_{\text{all}}={\text{Loss}}_{\text{pixel}}+{\text{Loss}}_{\text{region}}\end{eqnarray*}

where $F \left( C \right) $ represents frequency of the region belonging to class *C*, *DS* denotes data source, *A* symbolize extracted area, *A*_*y*_ indicates ground-truth of the region, *e*^*A*_*y*_^ is the exponential of the *A*_*y*_ used to calculate the probability distribution of regions across different classes and *e*^*A*_*k*_^ represents the exponential of the region logit corresponding to class *k* (*k* represents an index for the class in the label space *Y* and, Loss_pixel_ refers to pixel loss while Loss_region_ pertains to region loss.

#### Supervised contrastive learning network

We have devised supervised contrastive learning network (SCoLN) network to address the challenge of imbalanced data and enhance classification performance. This network adopts the classic UNet structure. In the encoder part, we have utilized a series of convolutional layers with varying kernel sizes to extract features from the input data. Each convolutional layer is followed BN and ReLU activation functions to enhance feature representation. Down-sampling is achieved using pooling to reduce the spatial dimensions while increasing the depth of feature maps. In the decoder we have employed bilinear interpolation is employed to reconstruct the original spatial resolution. The decoder mirrors the encoder’s architecture and incorporates skip connections to combine low-level and high-level features for precise localization. We have Incorporated contrastive learning into our UNet model to learns discriminative features for accurate image segmentation.

It takes the outputs generated by the CCGAN and focuses on identifying false positives and false negatives within the data. The culmination of our approach lies in the synthesis of these refined predictions. We have achieved this by adding the count of false negatives and subtracting the count of false positives predicted by the SCoLN from the CCGANs outputs.

This meticulous process ensures that our final semantic segmentation not only mitigates imbalanced data issues but also improves classification accuracy. During training, this network learns to capture both local and global features from the input representation, The learned representation can be used to improve the CCGAN’s weight updates, resulting in better image segmentation. The network is trained in a supervised contrastive learning framework, combining both the contrastive loss for similarity comparisons and a classification loss for the specific classification task as shown in Eq. (11).

(11) \begin{eqnarray*}{L}_{Scon} \left( {F}_{i},{F}_{p},{F}_{n} \right) =\log \nolimits \left( \frac{\exp \nolimits \left( simi \left( {F}_{i},{F}_{p} \right) \right) }{\sum _{m=1}^{N}\exp \nolimits \left( simi \left( {F}_{i},{F}_{m} \right) \right) } . \frac{\exp \nolimits \left( simi \left( {F}_{i},{F}_{n} \right) \right) }{\sum _{n=1}^{N}\exp \nolimits \left( simi \left( {F}_{i},{F}_{n} \right) \right) } \right) \end{eqnarray*}

where *F*_*i*_, *F*_*p*_, *F*_*n*_ represents reference features embedding, positive feature embedding and negative feature embedding respectively. *N* and *M* indicate positive and negative pairs. *simi* is employed as similarity metric.

In order to handle the miss-classification cost, the output by the generator and discriminator is passed to SCoLN. The network is utilized to delve into the intricate details of false predictions made by the CCGAN, specifically focusing on both positive and false negative cases.

The false negative error signifies the count of pixels that were erroneously labelled as background. Likewise, the false positive denotes the count of pixels that were inaccurately labelled as part of the foreground or region of interest. The SCoLN is trained to learn the CCGAN false prediction by focusing on false positives and negatives as shown in Eqs. (12) and (13).

(12) \begin{eqnarray*}& {C}_{ln}=con( \left( g-{L}_{Scon} \right) ,0,1)\end{eqnarray*}

(13) \begin{eqnarray*}& {C}_{lp}=con( \left( {L}_{Scon}-g \right) ,0,1)\end{eqnarray*}

where ***g*** and the *con* function is used to ensure that the loss is non-negative. 2*C*_*L*_ represents contrastive loss explain in the following.

#### Conditional contrastive loss

The conditional contrastive loss (2C loss) loss function aims to minimize the difference between the embeddings of the input sample and its corresponding mask while maximizing the difference between the input sample and other masks in the dataset. This helps in learning discriminative features for accurate image segmentation. This loss function can consider data-to-data and data-to-class association within the same class. The 2C loss for conditional image generation leverages contrastive learning to enhance both inter-class differentiation and intra-class consistency during image creation. By comparing features of generated images within and between classes, the conditional contrastive loss aims to produce realistic and visually cohesive images while sharpening class boundaries. The mathematical dynamics of the 2C loss function is given in Eq. (14).

(14) \begin{eqnarray*}2{C}_{L} \left( {I}_{i},{M}_{i};t \right) =-log \left( \frac{\exp \nolimits \left( l{ \left( {I}_{i} \right) }^{T}e({M}_{i})/t \right) +\sum _{j=1}^{x}{1}_{j={M}_{i}}.\exp \nolimits \left( l{ \left( {I}_{i} \right) }^{T}l({M}_{j})/t \right) }{\exp \nolimits \left( l{ \left( {I}_{i} \right) }^{T}e({M}_{i})/t \right) +\sum _{j=1}^{x}{1}_{j\not = i}.exp \left( l{ \left( {I}_{i} \right) }^{T}l({M}_{j})/t \right) } \right) .\end{eqnarray*}

The equation $2{C}_{L} \left( {I}_{i},{M}_{i};t \right) $ represents the supervised contrastive loss function used in image segmentation tasks where *I*_*i*_ denotes the input sample, *M*_*i*_ represents its corresponding mask, *t* is the temperature term used to adjust the SoftMax distribution and *x* indicate total number of samples. The term ${ \left( {I}_{i} \right) }^{T}$ refers to the transpose of the feature embedding $l \left( {I}_{i} \right) $, which encodes the features extracted from the input sample.

The expression $\exp \left( l{ \left( {I}_{i} \right) }^{T}e{M}_{i}/t \right) $ calculates the similarity between the input sample’s embedding vector and its associated ground-truth, where higher values indicate greater similarity. Similarly, $exp \left( l{ \left( {I}_{i} \right) }^{T}l({M}_{k})/t \right) $ measures the similarity between the embedding vector of the input sample and all other embedding, reflecting its dissimilarity in the feature space.

Previous studies (Dou et al., 2018; Shen et al., 2017) on conditional generative adversarial networks (CGANs) have suggested that incorporating an *L*_1_ loss term into the objective function enhance the model’s performance. This addition minimizes the absolute distance between the existing value and the predicted values. The *L*_1_ function considers differences in CNNs features between the ground-truth segmentation and the predicted segmentation, resulting in smoother boundaries and reduced noise. Mathematically shown in Eq. (15) as

(15) \begin{eqnarray*}{{L}_{l}}_{1} \left( G \right) ={E}_{a,n}{|}{|}{o}_{seg}-G \left( a,n \right) {|}{|}.\end{eqnarray*}

Adversarial loss for the segmentation task is estimated using Eq. (16) as

(16) \begin{eqnarray*}{m}_{seg} \left( D,G \right) =2{C}_{L}+{{L}_{l}}_{1} \left( G \right) .\end{eqnarray*}

The ultimate objective function of the proposed network, for semantic segmentation, is computed by the addition of the count of false negatives and subtraction of the count of false positives from the outputs of the adversarial model as shown in Eq. (17).

(17) \begin{eqnarray*}\text{loss}={m}_{seg} \left( D,G \right) -{C}_{lp}+{C}_{ln}.\end{eqnarray*}

### Evaluation measure

To evaluate the segmentation performance of the proposed model, we used widely recognized metrics, including intersection over union (IoU), dice similarity coefficient (DSC), Precision, Recall. These metrics are defined by the following Eqs. (18), (19), (20) and (21).

(18) \begin{eqnarray*}& \text{IoU}= \frac{2{T}^{P}}{2{T}^{P}+{F}^{P}+{F}^{N}} \end{eqnarray*}

(19) \begin{eqnarray*}& \text{DSC}= \frac{{T}^{P}}{{T}^{P}+{F}^{P}+{F}^{N}} \end{eqnarray*}

(20) \begin{eqnarray*}& \text{Precision}= \frac{{T}^{P}}{{T}^{P}+{F}^{P}} \end{eqnarray*}

(21) \begin{eqnarray*}& \text{Recall}= \frac{{T}^{P}}{{T}^{P}+{F}^{N}} .\end{eqnarray*}

### Implementation details

We have implemented the proposed model using the deep learning framework Keras and TensorFlow in Python 3.7. We utilized Matplotlib for presenting and visualizing the results. All dataset’s images were resized to a uniform size of 512 × 512 × 3 pixels. To normalize the pixel values and bring them into the range of 0 to 1, we applied Z-score normalization. Besides utilizing standard augmentation techniques like random cropping and rotation, we implement a contrast-constrained adaptive histogram equalization (CLANE) approach for pre-processing images within a specified region. This technique is effective in amplifying detailed features in MRI datasets, while simultaneously diminishing noise caused by artefacts.

Additionally, we employed random assignment to conduct a five-fold cross-validation. The model was trained from scratch on a computer equipped with 64 GB of RAM and an NVIDIA GeForce RTX 4060 Ti GPU. We employed the Adadelta optimizer with an initial learning rate of 0.001 and weight decay set to 1e−4 and temperature term *t* to 3. The training process spanned 450 epochs, incorporating an early stopping mechanism, and utilized a batch size of 32 for efficient training.

## Dataset

We have selected five different datasets for the evaluation of proposed CCGAN network. These datasets are: Bus2017, the Digital Database Thyroid Image (DDTI), LiTS MICCAI 2017, the ATLAS dataset, and BRATS 2015. A short description of each dataset is given below.

**BUS2017 dataset:** Digital mammography serves as a commonly employed initial screening method for detecting breast cancer. In this context, the BUS2017 dataset, meticulously assembled for breast cancer detection purposes, encompasses a collection of 163 ultrasound images. These images exhibit dimensions of 760 × 570 pixels. Among the dataset’s contents, 110 images depict benign lesions, encompassing 39 cases of fibroadenomas, six from other benign categories, and 65 instances of unspecified cysts. The remaining 53 ultrasound images vividly portray cancerous masses, primarily characterized by the presence of invasive ductal carcinomas.

**The Digital Database Thyroid Image (DDTI):** The DDTI dataset is a comprehensive collection of ultrasound images specifically created for thyroid cancer screening. DDTI primarily focuses on B-mode ultrasound imaging for thyroid cancer diagnosis, providing detailed diagnostic descriptions and annotations for each image. In total, the dataset includes 298 ultrasound images, with 270 of them featuring women and 29 featuring men.

**LiTS MICCAI 2017:** This dataset includes multiple images obtained from 260 patients who have been diagnosed with hepatocellular carcinoma. The dataset contains images of various sizes, which are used for the segmentation of both the tumour and liver.

**ATLAS dataset**: The ATLAS dataset consists of 90 MR images specifically focused on the liver, acquired from patients diagnosed with liver cancer. Within this dataset, you will find segmentation masks for both the liver and tumours, each corresponding to one of the 90 images.

**BRATS 2015**: The BRATS 2015 dataset comprises magnetic resonance images (MRI) focused on brain tumour detection. It encompasses a total of 220 MRI scans for high-grade gliomas (HGG) and 54 MRI scans for low-grade gliomas (LGG).

**Table 1:** The performance of deep learning models on MRI datasets after the integration of class-imbalancing mechanism except SCoLN.

**M** **odel**	**Io** **U**	**D** **SC**	**P** **recision**	**R** **ecall**	**Dataset**
UNet	0.86.5 ± 0.015	0.935 ± 0.012	0.925 ± 0.018	0.934 ± 0.016	**BUS2017**
FCN	0.754 ± 0.013	0.847 ± 0.011	0.816 ± 0.017	0.916 ± 0.015	
RCNN	0.865 ± 0.014	0.927 ± 0.012	0.917 ± 0.016	0.914 ± 0.014	
SegNet	0.826 ± 0.013	0.908 ± 0.011	0.897 ± 0.017	0.898 ± 0.015	
DeepLab-V3	0.897 ± 0.012	0.948 ± 0.010	0.948 ± 0.016	0.947 ± 0.014	
CGAN	0.896 ± 0.013	0.936 ± 0.011	0.945 ± 0.017	0.954 ± 0.015	
CCGAN	**0.921 ± 0.014**	**0.965 ± 0.012**	**0.956 ± 0.016**	**0.964 ± 0.017**	
**M** **odel**	**Io** **U**	**D** **SC**	**P** **recision**	**R** **ecall**	**DDTI**
UNet	0.784 ± 0.042	0.867 ± 0.023	0.735 ± 0.036	0.851 ± 0.064	
FCN	0.748 ± 0.028	0.744 ± 0.046	0.683 ± 0.072	0.696 ± 0.046	
RCNN	0.767 ± 0.014	0.855 ± 0.041	0.753 ± 0.064	0.848 ± 0.057	
SegNet	0.757 ± 0.095	0.845 ± 0.103	0.725 ± 0.87	0.718 ± 0.098	
DeepLab-V3	0.845 ± 0.015	0.895 ± 0.014	0.852 ± 0.058	0.855 ± 0.087	
CGAN	0.833 ± 0.097	0.884 ± 0.022	0.864 ± 0.053	0.863 ± 0.034	
CCGAN	**0.855 ± 0.24**	**0.896 ± 0.091**	**0.874 ± 0.044**	**0.872 ± 0.042**	
**M** **odel**	**Io** **U**	**D** **SC**	**Precision**	**R** **ecall**	
UNet	0.776 ± 0.062	0.673 ± 0.042	0.683 ± 0.055	0.844 ± 0.076	**LiTS** ** MICCAI 2017**
FCN	0.721 ± 0.056	0.655 ± 0.038	0.680 ± 0.053	0.741 ± 0.045	
RCNN	0.726 ± 0.054	0.647 ± 0.036	0.667 ± 0.051	0.755 ± 0.041	
SegNet	0.661 ± 0.050	0.550 ± 0.034	0.590 ± 0.049	0.682 ± 0.039	
DeepLab-V3	0.801 ± 0.065	0.763 ± 0.044	0.753 ± 0.059	0.833 ± 0.071	
CGAN	0.800 ± 0.063	0.742 ± 0.041	0.752 ± 0.056	0.832 ± 0.067	
CCGAN	**0.843 ± 0.068**	**0.786 ± 0.046**	**0.802 ± 0.063**	**0.884 ± 0.079**	
**M** **odel**	**Io** **U**	**D** **SC**	**P** **recision**	**R** **ecall**	
UNet	0.768 ± 1.2	0.666 ± 1.4	0.749 ± 1.3	0.830 ± 1.1	**ATLAS dataset**
FCN	0.745 ± 1.4	0.647 ± 1.5	0.646 ± 1.6	0.776 ± 0.9	
RCNN	0.778 ± 1.1	0.683 ± 1.3	0.738 ± 1.2	0.823 ± 1.2	
SegNet	0.754 ± 1.3	0.644 ± 1.6	0.699 ± 1.4	0.819 ± 1.3	
DeepLab-V3	0.796 ± 1.4	0.696 ± 1.1	0.757 ± 1.5	0.856 ± 1.0	
CGAN	0.787 ± 1.3	0.696 ± 1.2	0.767 ± 1.1	0.865 ± 1.3	
CCGAN	**0.799 ± 1.1**	**0.712 ± 1.5**	**0.780 ± 1.2**	**0.876 ± 1.1**	
**M** **odel**	**Io** **U**	**D** **SC**	**P** **recision**	**R** **ecall**	
UNet	0.922 ± 1.7	0.812 ± 1.2	0.903 ± 1.3	0.876 ± 1.4	**BRATS 2015**
FCN	0.909 ± 1.4	0.799 ± 1.6	0.874 ± 1.1	0.853 ± 1.5	
RCNN	0.907 ± 1.8	0.802 ± 1.9	0.905 ± 1.7	0.841 ± 1.8	
SegNet	0.891 ± 1.5	0.793 ± 1.1	0.881 ± 1.9	0.864 ± 1.6	
DeepLab-V3	0.943 ± 1.6	0.846 ± 1.4	0.914 ± 1.2	0.891 ± 1.1	
CGAN	0.932 ± 1.3	0.834 ± 1.7	0.911 ± 1.8	0.905 ± 1.9	
CCGAN	**0.950 ± 1.9**	**0.877 ± 1.2**	**0.943 ± 1.5**	**0.936 ± 1.3**	

**Notes.**

The best results are shown in bold.

## Result analysis and Discussion

Segmenting lesions in MRI scans is highly beneficial for automated cancer diagnosis, particularly in early detection and treatment phases. As presented in Table 1, our newly developed CCGAN achieves the highest dice similarity coefficient (DSC) at 0.965 ± 0.012 and the optimal Intersection over Union (IoU) at 0.921 ± 0.014, surpassing other advanced techniques for the BUS2017 dataset. In terms of precision, our CCGAN shows a notable improvement at 0.956 ± 0.016 on the test set, exceeding the 0.948 ± 0.016 attained by the closest rival, DeepLab-V3. Additionally, our approach surpasses traditional models like UNet and FCN in the DSC metric by 3.0% and 11.8%, respectively. Furthermore, when compared to the CGAN and SegNet networks, our method achieves performance enhancements of 2.9% and 5.9% in DSC metric, respectively. Our network demonstrates statistically significant advancements over others in terms of precision and recall. These findings indicate that our proposed system, incorporation of class-specific attention, RRM and supervised contrastive learning network, effectively captures more detailed context and finer features for lesion identification. Additionally, it demonstrates an ability to handle model bias resulting from class imbalance more effectively. As outlined in Table 1, our developed CCGAN achieves the highest DSC at 0.855 ± 0.24 and the IoU at 0.855 ± 0.24, surpassing other advanced deep learning methods on DDTI dataset. In terms of Precision and Recall CCGAN performance reaches 0.874 ± 0.044, 0.872 ± 0.042 respectively, exceeding the 0.874 ± 0.044, 0.872 ± 0.042 obtained by CGAN. Our network demonstrates statistically significant advancements over others in terms of precision and recall.

The proposed model also records the highest segmentation results on the, LiTS MICCAI 2017, ATLAS, and BRATS 2015 datasets, outshining other leading-edge methods. These comparative outcomes are also detailed in Table 1. Specifically, for the LiTS MICCAI 2017 dataset, our CCGAN model achieves a DSC of 0.786 ± 0.046, marking an enhancement of 2.3% and 4.4% over the next best models, Deeplabv3 and CGAN, respectively. Similarly, in the LiTS MICCAI 2017 dataset, our approach consistently surpasses other advanced architectures, showing a more significant improvement in liver tumour segmentation. In terms of Recall, Precision, IoU, and DSC metrics, all improvements made by CCGAN over other methods are statistically significant. This indicates that our method is effective in uniformly extracting features at the same level and integrating features across different levels. As a result, it leads to improved segmentation performance while addressing the negative impact of class imbalancing.

We have also utilized CCGAN for the task of liver segmentation from the ATLAS dataset. The comprehensive outcomes of this experiment are also listed in Table 1. The proposed CCGAN demonstrates top-tier performance across all measured metrics, achieving DSC of 0.712 ± 1.5, an IoU of 0.799 ± 1.1, a recall rate of 0.876 ± 1.1, and a precision rate of 0.780 ± 1.2. When compared with the outcomes achieved by established models such as UNet, SegNet, FCN, DeepLabv3, and RCNN, our CCGAN DSC score is significantly higher by margins of 4.6%, 6.8%,6.5%, 1.6%, and 2.9% respectively. These findings underscore that our model significantly enhances the effectiveness of segmentation.

We have also implemented our method for segmenting images from the BRATS 2015 datasets. The test outcomes are compared with those from five leading state-of-the-art (SOTA) models, as illustrated in Table 1. The comparison, based on metrics such as the overall DSC, IoU, recall, and precision, indicates that the results are of statistical significance. According to the data in the Table 1, our CCGAN demonstrates superior performance. This outcome further reinforces the idea that our CCGAN, enhanced with a contrastive learning approach, is advantageous for segmenting medical images.

To gain a better understanding of the performance improvements, we have analysed the achieved performance of deep learning models without considering the class-imbalancing mechanism on the MRI dataset segmentation. Table 2 reveals a significant decrease in the performance of the baseline state-of-the-art models for these datasets.

**Table 2:** The performance of deep learning models before integrating class-imbalancing mechanism on MRI datasets.

**M** **odel**	**Io** **U**	**D** **SC**	**P** **recision**	**R** **ecall**	**Dataset**
UNet	0.836 ± 0.040	0.906 ± 0.031	0.908 ± 0.053	0.882 ± 0.042	**BUS2017**
FCN	0.745 ± 0.027	0.820 ± 0.023	0.773 ± 0.045	0.884 ± 0.050	
RCNN	0.843 ± 0.055	0.916 ± 0.053	0.886 ± 0.031	0.896 ± 0.039	
SegNet	0.834 ± 0.036	0.876 ± 0.054	0.876 ± 0.038	0.886 ± 0.056	
DeepLab-V3	0.832 ± 0.028	**0.928 ± 0.035**	**0.919 ± 0.025**	**0.929 ± 0.048**	
CGAN	**0.846 ± 0.053**	0.882 ± 0.046	0.915 ± 0.025	0.925 ± 0.042	
**M** **odel**	**Io** **U**	**D** **SC**	**P** **recision**	**R** **ecall**	
UNet	0.748 ± 0.083	0.853 ± 0.071	0.725 ± 0.086	0.838 ± 0.068	**DDTI**
FCN	0.734 ± 0.076	0.731 ± 0.064	0.688 ± 0.057	0.689 ± 0.062	
RCNN	0.724 ± 0.098	0.852 ± 0.086	0.726 ± 0.098	0.824 ± 0.077	
SegNet	0.764 ± 0.094	0.837 ± 0.078	0.707 ± 0.064	0.690 ± 0.059	
DeepLab-V3	**0.846 ± 0.057**	**0.868 ± 0.064**	**0.848 ± 0.059**	**0.848 ± 0.085**	
CGAN	0.835 ± 0.078	0.867 ± 0.067	0.833 ± 0.058	0.842 ± 0.069	
**M** **odel**	**Io** **U**	**D** **SC**	**P** **recision**	**R** **ecall**	
UNet	0.733 ± 0.066	0.666 ± 0.037	0.635 ± 0.058	0.828 ± 0.087	**LiTS** ** MICCAI 2017**
FCN	0.695 ± 0.080	0.635 ± 0.067	0.656 ± 0.022	0.735 ± 0.066	
RCNN	0.678 ± 0.044	0.608 ± 0.043	0.661 ± 0.040	0.737 ± 0.064	
SegNet	0.634 ± 0.065	0.540 ± 0.047	0.574 ± 0.059	0.642 ± 0.085	
DeepLab-V3	**0.766 ± 0.084**	**0.695 ± 0.038**	**0.742 ± 0.049**	**0.834 ± 0.062**	
CGAN	0.743 ± 0.044	0.647 ± 0.079	0.731 ± 0.056	0.830 ± 0.080	
**M** **odel**	**Io** **U**	**D** **SC**	**P** **recision**	**R** **ecall**	
UNet	0.711 ± 1.3	0.645 ± 1.2	0.684 ± 1.5	0.775 ± 1.2	**ATLAS dataset**
FCN	0.705 ± 1.4	0.623 ± 1.4	0.675 ± 1.3	0.765 ± 1.5	
RCNN	0.691 ± 1.2	0.586 ± 1.3	0.647 ± 1.4	0.736 ± 1.1	
SegNet	0.714 ± 1.5	0.638 ± 1.4	0.669 ± 1.2	0.776 ± 1.3	
DeepLab-V3	**0.736 ± 1.3**	**0.639 ± 1.5**	**0.737 ± 1.4**	**0.786 ± 1.1**	
CGAN	0.732 ± 1.4	0.622 ± 1.3	0.733 ± 1.5	0.776 ± 1.2	
**M** **odel**	**Io** **U**	**D** **SC**	**P** **recision**	**R** **ecall**	
UNet	0.849 ± 1.2	0.777 ± 1.7	0.843 ± 1.6	0.828 ± 1.7	**BRATS 2015**
FCN	0.843 ± 1.3	0.763 ± 1.5	0.825 ± 1.7	0.774 ± 1.3	
RCNN	0.850 ± 1.6	0.753 ± 1.6	0.853 ± 1.5	0.773 ± 1.5	
SegNet	0.835 ± 1.4	0.753 ± 1.6	0.847 ± 1.3	0.780 ± 1.7	
DeepLab-V3	**0.886 ± 1.6**	**0.762 ± 1.3**	0.863 ± 1.3	0.852 ± 1.5	
CGAN	0.873 ± 1.9	0.758 ± 1.5	**0.876 ± 1.5**	**0.855 ± 1.8**	

**Notes.**

The best results are shown in bold.

### Comparison with other attention mechanism

We have compared the performance of our approach with other state-of-the-art attention techniques. For a fair comparison, the attention mechanisms from the literature are employed at the same position in the encoder of the generator in CGAN and DeepLab-V3. Table 3 compares the outcomes of the attention techniques for different MRI datasets. The results demonstrate that the class-attention scheme performs effectively well to detect minority class that is lesions as compared with its counterparts for MRI datasets. Table 3 demonstrates how the suggested attention approach outperforms other techniques in terms of IoU, DSC Precision, and Recall. Compared to alternative approaches, our proposed method demonstrates competitive performance, achieving improvements of 3.2%, 2.4%, and 1.2%, in IoU compared to soft-attention, spatial, and hybrid attention mechanisms for DeepLab-V3 on the BRATS 2015 dataset. Table 3 also highlights improvements in various metrics for the LiTS MICCAI 2017 dataset. The class-wise attention mechanism has proven to be highly effective in identifying intra-class variation, inter-class similarity, and addressing class imbalance. The other employed approaches demonstrate significant performance on the MRI dataset; however, they still encounter challenges in handling underrepresented classes.

**Table 3:** A comparison of various attention mechanisms for deep learning models.

**M** **odel**	**Io** **U**	**D** **SC**	**P** **recision**	**R** **ecall**	**Dataset**
DeepLab-V3+soft attention	0.903 ± 1.3	0.795 ± 0.99	0.879 ± 1.8	0.864 ± 1.2	**BRATS 2015**
DeepLab-V3+ spatial	0.911 ± 1.5	0.813 ± 1.2	0.885 ± 1.8	0.868 ± 1.5	
DeepLab-V3+hybrid	0.923 ± 1.5	0.824 ± 1.4	0.895 ± 1.6	0.875 ± 1.7	
DeepLab-V3+class-specific	**0.935 ± 1.1**	**0.830 ± 1.7**	**0.901 ± 1.2**	**0.878 ± 1.9**	
CCGAN+ soft attention	0.919 ± 1.2	0.814 ± 1.6	0.903 ± 1.5	0.856 ± 1.7	
CCGAN+ spatial	0.928 ± 1.6	0.826 ± 1.5	0.919 ± 1.5	0.876 ± 1.9	
CCGAN+ hybrid	0.931 ± 1.3	0.837 ± 1.4	0.921 ± 1.1	0.911 ± 1.2	
CCGAN+ class-specific	**0.936 ± 1.5**	**0.842 ± 1.5**	**0.925 ± 1.8**	**0.916 ± 1.3**	
					**LiTS** ** MICCAI 2017**
DeepLab-V3+soft attention	0.762 ± 0.066	0.664 ± 0.042	0.726 ± 0.081	0.802 ± 0.057	
DeepLab-V3+ spatial	0.775 ± 0.089	0.687 ± 0.048	0.724 ± 0.091	0.809 ± 0.069	
DeepLab-V3+hybrid	0.788 ± 0.072	0.718 ± 0.055	0.738 ± 0.073	0.818 ± 0.083	
DeepLab-V3+class-specific	**0.792 ± 0.093**	**0.735 ± 0.065**	**0.742 ± 0.038**	**0.821 ± 0.041**	
CCGAN+ soft attention	0.779 ± 0.068	0.722 ± 0.072	0.764 ± 0.098	0.837 ± 0.093	
CCGAN+ spatial	0.786 ± 0.053	0.730 ± 0.087	0.774 ± 0.063	0.855 ± 0.073	
CCGAN+ hybrid	0.816 ± 0.084	0.762 ± 0.044	0.784 ± 0.098	0.862 ± 0.082	
CCGAN+ class-specific	**0.824 ± 0.058**	**0.764 ± 0.098**	**0.784 ± 0.054**	**0.865 ± 0.045**	

**Notes.**

The best results are shown in bold.

**Table 4:** The impact of different modules on the performance of CCGAN.

**Model**	**IoU**	**DSC**	**Precision**	**Recall**	
CCGAN	0.924 ± 1.6	0.830 ± 1.7	0.910 ± 1.8	0.900 ± 1.5	**BRATS 2015**
CCGAN + class-specific	0.936 ± 1.5	0.842 ± 1.5	0.925 ± 1.8	0.916 ± 1.3	
CCGAN + class-specific + RRM	0.941 ± 1.7	0.855 ± 1.8	0.930 ± 1.5	0.923 ± 1.9	
CCGAN + class-specific + RRM+SCoLN	**0.950 ± 1.9**	**0.877 ± 1.2**	**0.943 ± 1.5**	**0.936 ± 1.3**	
CCGAN	0.803 ± 0.033	0.747 ± 0.021	0.755 ± 0.085	0.830 ± 0.25	**LiTS** ** MICCAI 2017**
CCGAN+ class-specific	0.824 ± 0.049	0.768 ± 0.015	0.783 ± 0.052	0.864 ± 0.017	
CCGAN + class-specific + RRM	0.828 ± 0.057	0.780 ± 0.032	0.795 ± 0.061	0.871 ± 0.048	
CCGAN + class-specific + RRM+SCoLN	**0.843 ± 0.068**	**0.786 ± 0.046**	**0.802 ± 0.063**	**0.884 ± 0.079**	

**Notes.**

The best results are shown in bold.

### Impact of the region rebalancing module and supervised contrastive learning-based network

The Table 4 below, provides a comprehensive analysis of the impact of the region rebalancing mechanism (RRM) and SCoLN on the performance of the CCGAN for both the BRATS 2015 and LiTS MICCAI 2017 datasets. The results demonstrate the effectiveness of both modules in addressing the class imbalance problem prevalent in highly imbalanced datasets. Table 4 showcases that the proposed class-specific attention mechanism enhances model performance by approximately 1.2% in terms of DSC and 1.2% in terms of IoU. Additionally, the incorporation of RRM and SCoLN significantly improves model performance by mitigating the impact of class imbalance issues in MRI datasets for deep learning-based models.

### Visualization and qualitative result analysis

**Figure 3:**
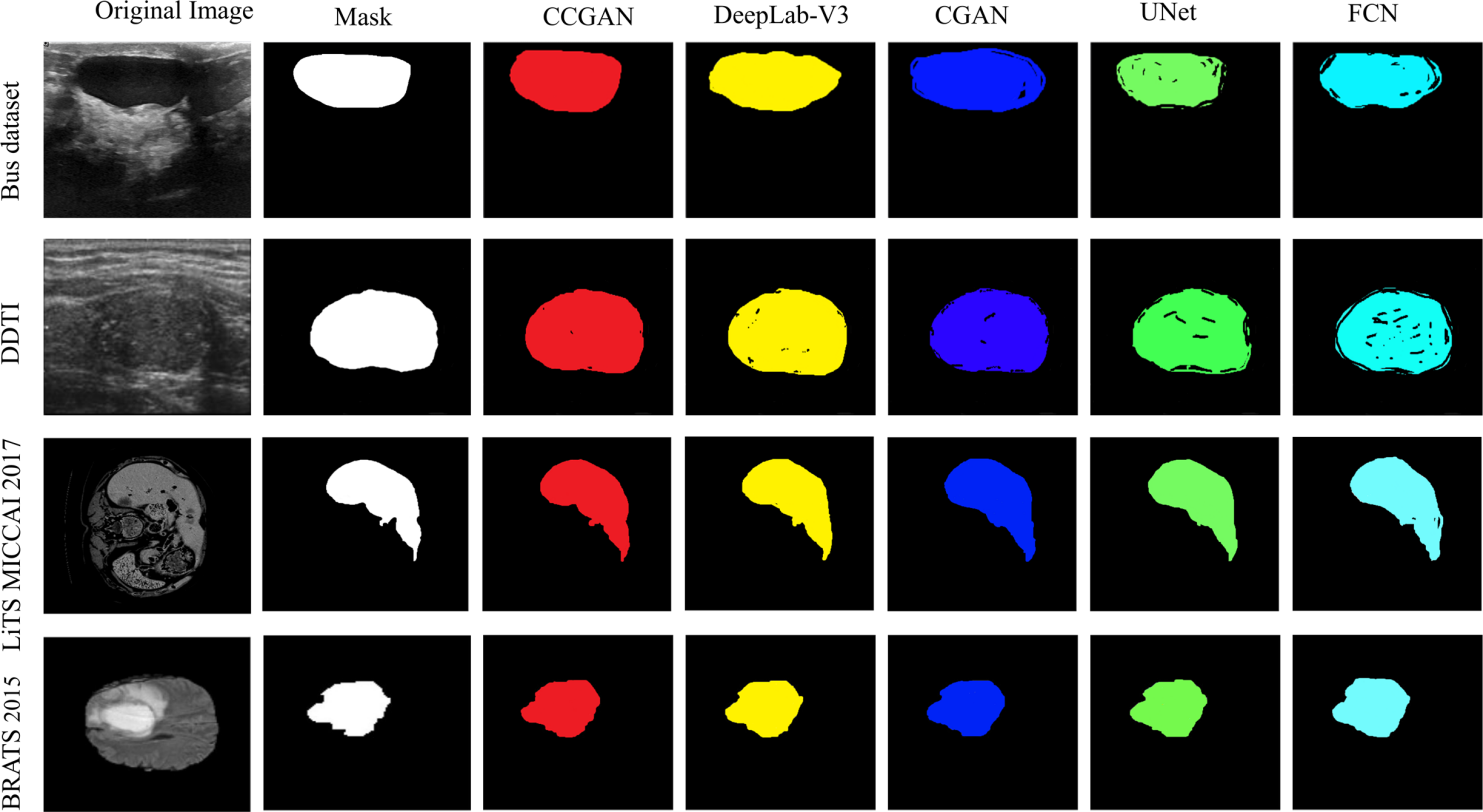
Visual comparison of the performance of various deep learning models. Black denotes mis-segmented regions.

Figure 3 display few instances of segmentation images from different MRI dataset created by our CGAN and other top-performing methods. The results clearly demonstrate that our approach excels in precisely highlighting objects with their accurate location and details. At the same time, it more effectively reduces the visibility of similar background areas and noise. Observing the first to fourth rows of Fig. 3, it is noticeable that some methods only partially segment lesions and inadvertently include irrelevant non-foreground elements. In stark contrast, our method shows exceptional performance. It accurately pinpoints target regions, aided by efficient contextual representations. Overall, the results indicate that our CGAN adeptly explores context at a uniform level and leverages the interrelations among features at different levels to enhance feature representation. This underlines CGAN’s capacity to handle intricate structures and correct inaccuracies.

## Conclusion

In this research, we have developed a novel deep learning architecture namely CCGAN to tackle the problem of class imbalancing in MRI dataset widely used for segmentation problem. The proposed model incorporates a class-specific attention mechanism, a region rebalancing module, and a discriminator and a supervised contrastive learning-based network to enhance output further. The class-specific attention is designed to selectively learn more relevant information effectively from input data. To enhance the model’s ability to address class imbalance, a region-level rebalancing mechanism is introduced, which enhance model robustness by distributing features equally across different regions of input images and resulting in improved segmentation. We integrated the RRM and class-specific attention module into various deep learning models. The CCGAN is supplemented by an additional discriminator, known as SCoLN, which is trained to predict false negative and false positive masks generated by the CCGAN’s generator. The analysis of results reveals a significant improvement in the segmentation performance of the baseline deep learning models for highly imbalanced MRI datasets.

## Supplemental Information

10.7717/peerj-cs.2064/supp-1 Supplemental Information 1 Python code for the conditional contrastive GAN

## Additional Information and Declarations

### Data Availability

The following information was supplied regarding data availability: The code is available in the Supplementary File. The data associated with this work are available at: - Breast Ultrasound Dataset (BUS2017), http://www2.docm.mmu.ac.uk/STAFF/m.yap/dataset.php
- Digital Database Thyroid Image (DDTI), https://www.kaggle.com/datasets/dasmehdixtr/ddti-thyroid-ultrasound-images
- Liver and Liver Tumor Segmentation (LiTS MICCAI 2017), https://www.kaggle.com/datasets/andrewmvd/lits-png
- A Tumor and Liver Automatic Segmentation (ATLAS) dataset, https://atlas-challenge.u-bourgogne.fr/
- Brain Tumor Image Segmentation Benchmark (BRATS 2015), https://paperswithcode.com/dataset/brats-2015-1.
